# Knowledge, Attitudes, and Practice Patterns Relating to Sexual Dysfunction Among Urologists and Andrologists in China

**DOI:** 10.1001/jamanetworkopen.2022.50177

**Published:** 2023-01-12

**Authors:** Dongdong Tang, Yuyang Zhang, Wei Zhang, Guanjian Li, Hao Geng, Hui Jiang, Xiansheng Zhang

**Affiliations:** 1 Reproductive Medicine Center, Department of Obstetrics and Gynecology, the First Affiliated Hospital of Anhui Medical University, Hefei City, China; 2 NHC Key Laboratory of Study on Abnormal Gametes and Reproductive Tract, Anhui Medical University, Hefei City, China; 3 Key Laboratory of Population Health Across Life Cycle, Anhui Medical University, Ministry of Education of the People’s Republic of China, Hefei City, China; 4 Department of Urology, the First Affiliated Hospital of Anhui Medical University, Hefei City, China; 5 Institute of Urology, the First Affiliated Hospital of Anhui Medical University, Hefei City, China; 6 Anhui Province Key Laboratory of Genitourinary Diseases, Anhui Medical University, Hefei City, China; 7 Department of Urology, Peking University First Hospital, Beijing, China; 8 Institute of Urology, Peking University, Beijing, China; 9 Andrology Center, Peking University First Hospital, Beijing, China

## Abstract

**Importance:**

Although sexual function is an indispensable part of overall human health, both male and female sexual dysfunction have been poorly addressed in China.

**Objective:**

To explore the self-reported knowledge, attitudes, and practice patterns related to sexual dysfunction among urologists and andrologists in China.

**Design, Setting, and Participants:**

This survey study enrolled urologists and andrologists from various regions in China. Urologists and andrologists from 100 selected medical centers were invited to participate in the survey. Responses from eligible urologists and andrologists were analyzed from July 20 to 30, 2022.

**Main Outcomes and Measures:**

The primary outcomes were the respondents’ basic characteristics and knowledge, attitudes, and practice patterns related to sexual dysfunction for both male and female patients.

**Results:**

Among 1687 urologists and andrologists (749 [98.7%] male; 375 participants [49.4%] aged 36-50 years) invited to participate, 759 were eligible, with 395 participants (52.0%) determined to have passing knowledge on diagnosis and treatment for male and female sexual dysfunction (defined as a score of 6 or more points) and 523 participants (68.9%) responding with positive attitudes, including interested in providing sex counseling and managing sexual issues for male and female patients. Moreover, of 395 individuals with passing knowledge, 289 participants (73.2%) were interested in providing sex counseling or sexual function care to patients; 336 participants (85.1%) thought they should routinely screen the spouse for sexual dysfunction when the partner was diagnosed with a sexual dysfunction. Among individuals with passing knowledge, 232 participants (58.7%) reported knowledge on the guidelines for diagnosis and treatment of premature ejaculation and 162 participants (41.0%) reported knowledge on the guidelines for the diagnosis and treatment of erectile dysfunction, which were significantly higher than rates among 364 participants with limited knowledge (premature ejaculation: 140 participants [38.5%]; erectile dysfunction: 78 participants [21.4%]). Only a few urologists and andrologists could manage female sexual dysfunction following guidelines, although the proportions were higher in the group with passing knowledge (38 participants [9.6%]) compared with those with less knowledge (5 participants [1.4%]). Additionally, most participants felt confident to manage male sexual issues (569 participants [75.0%] reporting often or almost always), while most of them were not so confident on female sexual issues (274 participants [36.1%] reporting often or almost always), mainly due to lacking knowledge in this field (518 participants [68.2%]).

**Conclusions and Relevance:**

The findings of this survey study suggest that urologists and andrologists in China lacked knowledge on sexual dysfunction, which in turn was associated with their attitudes and clinical practice patterns, especially for female sexual dysfunction. More training on sexual dysfunction should be undertaken to improve this situation.

## Key Points

**Question:**

What are the urologists’ and andrologists’ self-reported knowledge, attitudes, and practice patterns regarding sexual dysfunction?

**Findings:**

In this survey study among 759 urologists and andrologists in China, a significant number of respondents reported lacking knowledge on sexual dysfunction, which was associated with their attitudes and clinical practice patterns, especially for female sexual dysfunction.

**Meaning:**

These findings suggest that improving urologists’ and andrologists’ knowledge of sexual dysfunction could be beneficial to positive attitudes and clinical practice patterns for both male and female patients.

## Teaser

This survey study explores the knowledge, attitudes, and practice patterns related to sexual dysfunction among urologists and andrologists in China.

## Introduction

Sexual health, defined as a continuum of physical, psychological, and sociocultural well-being associated with sexuality, is an indispensable part of overall human health and quality of life, encompassing sexual function, sexually transmitted infections, psychological distress, and more.^1^ Sexual function is an important aspect of overall sexual health, and sexual dysfunction can have a devastating effect on sexual health. Sexual dysfunction refers to a problem that occurs during the sexual response cycle that prevents the individual from experiencing satisfaction from sexual activity.^2^ A substantial proportion of men and women worldwide experience various sexual dysfunctions, and studies suggest that prevalence is increasing.^2,3^ For males, erectile dysfunction (ED) and premature ejaculation (PE) are the most common sexual disorders.^4^ Data from the Massachusetts Male Aging Study^5^ indicate an ED prevalence of 52% in men aged older than 40 years. Additionally, the PE Prevalence and Attitudes survey,^6^ which enrolled more than 12 000 participants, suggested a PE prevalence of 22.7% over 24 years. For females, a worse situation has been reported. Approximately 43% of women in the US experience female sexual dysfunction,^7^ while in China, female sexual dysfunction is even more prevalent, possibly associated with China’s relatively conservative sexual culture.^8,9^ An internet-based study conducted by Du et al^10^ found the prevalence of female sexual dysfunction in China was 60.2%.

However, despite the high prevalence of sexual dysfunction, it continues to remain underdiagnosed and undertreated, especially female sexual dysfunction. A cross-sectional study conducted in Beijing, China,^9^ enrolling 4697 consecutive female participants, reported that the prevalence of female sexual dysfunction was 63.3%, while approximately 50% of female participants with sexual dysfunction had not been diagnosed or treated. Among the barriers related to high prevalence and low consultation rate of sexual dysfunction are not only patients’ embarrassment and feelings of indifference at seeking help but also physicians’ lack of knowledge on sexual dysfunction and inability to provide adequate help.^11^

Despite the fact that many andrology centers have been established in China and urologists and andrologists have been paying more attention to sexual function, there is still much room for improvement in physician training on sexual dysfunction in China, especially regarding female sexual dysfunction. Female sexual dysfunction was previously considered a psychological disorder, and patients sought help from mental health professionals instead of clinical physicians.^2^ Furthermore, this situation may be a global phenomenon. For example, an investigation on resident training in female sexual dysfunction conducted in the US found that only 37% of urologists were trained to screen for female sexual dysfunction, and only 24% of them were encouraged to screen for female sexual dysfunction.^12^ Similarly, a survey for residents in urology, obstetrics, and gynecology in Canada^13^ found that only 45.7% of residents received training on female sexual dysfunction during medical school. Additionally, there are few specialized sexual medicine physicians or sexologists in China who could consult on sexual dysfunction for both male and female patients. Male sexual dysfunction is mainly managed by urologists and andrologists, while female sexual dysfunction is mainly managed by obstetricians and gynecologists or psychologists.^14^ However, the associations between female sexual dysfunction and male sexual dysfunction within relationships have been well documented over the last decade.^8,15^ Consequently, urologists and andrologists should routinely screen sexual function of the female partners of male patients with sexual dysfunction to improve therapeutic outcomes.^16^ Nevertheless, it is difficult to manage sexual dysfunction when there is a lack of training or knowledge on the subject, especially for female sexual dysfunction.

To our knowledge, no studies have explored the knowledge, attitudes, and practice patterns of urologists and andrologists in China with regard to male and female sexual dysfunction. Therefore, we conducted this survey study to explore these topics and to assess the associations between these aspects related to sexual dysfunction in urologists and andrologists in China.

## Methods

This survey study was approved by the ethics committee of the First Affiliated Hospital of Anhui Medical University, and all participants provided electronic written informed consent to participate in the survey. This study is reported following the American Association for Public Opinion Research (AAPOR) reporting guideline.

### Study Design

A 2-stage quota sampling method was applied to achieve a nationally representative sample with respect to the regions of China, including midwestern and eastern regions and rural and urban areas. The first step was the sampling of hospitals, and the second step was sampling physicians. Eventually, a total of 1600 physicians were invited to participant in this survey from the 100 selected hospitals, including 500 from midwestern urban areas, 500 from eastern urban areas, 400 from midwestern rural areas, and 200 from eastern rural areas (eAppendix in Supplement 1). Additionally, 1 or 2 physicians or managers from each institution were designated as liaisons for the survey to ensure effective 2-way communication.

From May to July 2022, the survey was administered to a cohort of full-time urologists and andrologists working in all invited hospitals using a secure, anonymous, and web-based research database (Wenjuan Star, free version; Changsha Ranxing Information Technology). Urologists and andrologists were identified using the staff list and liaisons of their institutions. The invitations were sent directly to all liaison practitioners within each institution via internal instant messaging software or emails.

The survey was accompanied by an invitation letter explaining the topic and purpose of the study and a link to the web-based questionnaire. It was first distributed on May 8, 2022. The invitation letter stated that the goal of the survey was to understand the sexual health clinical practices of urologists and andrologists in China. To increase response rates, electronic reminders were sent to nonresponders 3 and 7 days after the initial invitation.

### Survey Instrument

The initial survey was directed by the China Sexology Association and was created based on the International Society of Sexual Medicine’s guidelines for PE,^17^ the American Urological Association’s guidelines for ED,^18^ the American College of Obstetricians and Gynecologists practice bulletin (No. 119/213),^19^ and American Psychiatric Association’s *Diagnostic and Statistical Manual of Mental Disorders* (Fifth Edition) (*DSM-5*).^20^ Before the formal investigation, a preliminary questionnaire was tested by a total of 15 urologists and andrologists to assess its flow, content, validity, and clinical utility. The survey was then modified by the initiator based on feedback from the pilot test; pilot participants were excluded from the formal investigation. Finally, 29 items were finalized in the survey (eAppendix in Supplement 1), including demographic data (8 items), knowledge (8 items, including 6 male sexual dysfunction–related questions [K1-K6] and 2 female sexual dysfunction–related questions [K7-K8]), attitude (5 items), and practice patterns (4 items, including 2 for male sexual dysfunction [P1-P2] and 2 for female sexual dysfunction [P3-P4]) related to sexual dysfunction, as well as their confidence and main difficulties in addressing and managing male and female sexual dysfunction (4 items). To quantitatively explore the associations among knowledge, attitudes, and practice patterns related to sexual dysfunction, a 10-point system was adopted to score for the knowledge and attitudes sections, and a 60% threshold for the knowledge and attitudes tests was adopted, combining with our previous study.^14^ The detailed scoring method is described in the eAppendix in Supplement 1. After finalizing the questionnaire, another 20 urologists and andrologists were invited to complete the survey to assess minimum time to complete the questionnaire, and the minimum time was determined to be 2 minutes.

### Study Population

Participants who met all of the following criteria were included in the analysis: licensed physicians specializing in urology and andrology, providing direct patient care in the eligible specialties, and completing all items in our survey using more than 2 minutes. Participants were excluded from our study if they shared the same IP address, did not complete the questionnaire, completed the questionnaire in less than 2 minutes, or were graduate students or interns. Initially, 1687 urologists and andrologists were invited to participate in this survey, and 844 participants responded. However, 85 respondents were excluded, including 58 individuals who answered the questionnaire in less than 2 minutes, 16 individuals who no longer worked in clinical practice, and 11 individuals who were graduate students or interns. Eventually, a total of 759 eligible urologists and andrologists (response rate, 45.0%) were included in the final calculation and analysis.

### Statistical Analysis

All statistical analyses were conducted using SPSS software version 18.0 (SPSS), and statistical significance was defined as a 2-sided *P* < .05. Categorical variables are shown using descriptive statistics, including frequency counts and percentages. We used χ^2^ test or Fisher exact test for categorical variables as appropriate to compare differences between the group with and without abundant knowledge on sexual dysfunction. Data were analyzed from July 20 to 30, 2022.

## Results

A total of 759 eligible urologists and andrologists were included in the final analyses, with a response rate of 45.0%. Among all respondents, 749 (98.7%) were male and 10 (1.3%) were female, and 375 participants (49.4%) were aged 36 to 50 years. Most participants (695 participants [91.6%]) specialized in Western medicine, while only 64 participants (8.4%) specialized in traditional Chinese medicine. In the knowledge test, 395 urologists and andrologists (52.0%) scored at least 6 points of a total score of 10 points, and the 759 eligible participants were classified into 2 groups with the cutoff value of 6 points. Individuals scoring at least 6 points were classified as having passing knowledge on the diagnosis and treatment for male and female sexual dysfunction, while scoring fewer than 6 indicated lacking knowledge on these aspects. No statistically significant differences were found between groups with respect to age, sex, education level, professional title, time in practice, subspecialty, or proportion of patients in their practice with sexual dysfunction (Table 1). There was a significant difference in practice setting among participants who scored fewer than 6 points vs those who scored 6 or more points (tertiary hospitals: 272 participants [74.7%] vs 320 participants [81.0%]; secondary hospitals: 92 participants [25.3%] vs 75 participants [19.0%]; *P* = .04). For the attitude test, the same pass threshold was qualified at scoring 6 or more points of the total score of 10 points, and 523 urologists and andrologists (68.9%) received a passing score on the attitude test, including questions regarding interest in providing sex counseling and managing sexual health for male and female patients. No statistically significant differences were found between groups based on attitude scores. The detailed demographic characteristics are presented in Table 1.

**Table 1.** Demographic Characteristics of Respondents

Characteristic	Respondents, No. (%)			*P* value^a^	Knowledge score, No. (%)		*P* value^a^
	Total	Attitude score					
		<6 Points (n = 236)	≥6 Points(n = 523)		<6 Points (n = 364)	≥6 Points (n = 395)	
Age, y							
20-35	262 (34.5)	79 (33.5)	183 (35.0)	.91	140 (38.5)	122 (30.9)	.06
36-50	375 (49.4)	119 (50.4)	256 (48.9)		164 (45.1)	211 (53.4)	
>50	122 (16.1)	38 (16.1)	84 (16.1)		60 (16.5)	62 (15.7)	
Sex							
Male	749 (98.7)	234 (99.2)	515 (98.5)	.73	359 (98.6)	390 (98.7)	.90
Female	10 (1.3)	2 (0.8)	8 (1.5)		5 (1.4)	5 (1.3)	
Education level							
≤Bachelor’s degree	356 (46.9)	110 (46.6)	246 (47.0)	.50	176 (48.4)	180 (45.6)	.60
Master’s degree	309 (40.7)	92 (39.0)	217 (41.5)		147 (40.4)	162 (41.0)	
Doctorate	94 (12.4)	34 (14.4)	60 (11.5)		41 (11.3)	53 (13.4)	
Practice setting							
Tertiary hospital	592 (78.0)	180 (76.3)	412 (78.8)	.44	272 (74.5)	320 (81.0)	.04
Secondary hospital	167 (22.0)	56 (23.7)	111 (21.2)		92 (25.3)	75 (19.0)	
Professional title^b^							
Senior	356 (46.9)	110 (46.6)	246 (47.0)	.90	160 (44.0)	196 (49.6)	.10
Medium	259 (34.1)	79 (33.5)	180 (34.1)		124 (34.1)	135 (34.2)	
Junior	144 (19.0)	47 (19.9)	97 (19.0)		80 (22.0)	64 (16.2)	
Time in practice, y							
<5	152 (20.0)	53 (22.5)	99 (18.9)	.49	82 (22.5)	70 (17.7)	.24
5-15	319 (41.9)	94 (39.8)	225 (43.0)		150 (41.2)	169 (42.8)	
>15	288 (37.9)	89 (37.7)	199 (38.0)		132 (36.3)	156 (39.5)	
Subspecialty							
Traditional Chinese medicine	64 (8.4)	17 (7.2)	47 (9.0)	.43	27 (7.4)	37 (9.4)	.33
Western medicine	695 (91.6)	219 (92.8)	476 (91.0)		337 (92.6)	358 (90.6)	
Patients in practice with sexual dysfunction, %							
<25	462 (60.9)	152 (64.4)	310 (59.3)	.42	235 (64.6)	227 (57.5)	.19
25-50	230 (30.3)	63 (26.7)	167 (31.9)		98 (26.9)	132 (33.4)	
50-75	49 (6.5)	14 (5.9)	35 (6.7)		24 (6.6)	25 (6.3)	
>75	18 (2.4)	7 (3.0)	11 (2.1)		7 (1.9)	11 (2.8)	

^a^ χ^2^ test or Fisher exact test for categorical variables was used as appropriate.

^b^ In China, *junior* refers to residents, *medium* refers to attending physicians, and *senior* refers to associate chief physicians and chief physicians.

To further analyze the association between attitudes and knowledge, we compared the attitude scores and specific answers between groups with knowledge scores of 6 or more points vs fewer than 6 points. In the group with fewer than 6 points in the knowledge test, 234 participants (64.3%) had passing scores in the attitude questions, which was significantly lower than that in the group with knowledge scores of 6 or more points (289 participants [73.2%]; *P* = .008). We then compared distribution of answers to each attitude question between groups, and it was found that only the answers to the first attitude question, suggesting a patient’s sexual life is private and should not be interfered with, showed no difference between, with both groups having more than 50% of agreement. The remaining answers for the other 4 questions showed significant differences, including being interested in providing sex counseling and managing sexual issues for male and female patients and considering female sexual issues as priority diseases (Table 2). Of 395 individuals with passing knowledge, 313 participants (79.2%) were interested in providing sex counseling or sexual function care to patients; 336 participants (85.1%) thought they should routinely screen the spouse for sexual dysfunction when the partner was diagnosed with a sexual dysfunction.

**Table 2.** Associations Between Attitude and Knowledge Among Respondents

Question	Knowledge score, No, (%)		*P* value^a^	Overall *P *value
	<6 Points (n = 364)	≥6 Points (n = 395)		
Attitude score, No. (%)				
<6 Points	130 (35.7)	106 (26.8)	.008	NA
≥6 Points	234 (64.3)	289 (73.2)		
Sexual life is private and should not be interfered with				
Totally agree	95 (26.1)	123 (31.1)	.13	.58
Agree	93 (25.5)	101 (25.6)	>.99	
Neither agree nor disagree	83 (22.8)	78 (19.7)	.27	
Disagree	68 (18.7)	69 (17.5)	.66	
Completely disagree	25 (6.9)	24 (6.1)	.66	
I am interested in providing sex counseling or sexual health care to patients				
Totally agree	89 (24.5)	133 (33.7)	.007	.001
Agree	155 (42.6)	180 (45.6)	.41	
Neither agree nor disagree	101 (27.7)	64 (16.2)	<.001	
Disagree	15 (4.1)	10 (2.5)	.22	
Completely disagree	4 (1.1)	8 (2.0)	.31	
I do not think female sexual health issues are important or priority diseases				
Totally agree	9 (2.5)	12 (3.0)	.64	.01
Agree	28 (7.7)	32 (8.1)	.84	
Neither agree nor disagree	69 (19.0)	48 (12.2)	.01	
Disagree	185 (50.8)	188 (47.6)	.37	
Completely disagree	73 (20.1)	115 (29.1)	.004	
Screening and managing female sexual health issues are more the responsibility of obstetricians and gynecologists than of urologists or sexologists				
Totally agree	14 (3.8)	14 (3.5)	.83	.04
Agree	56 (15.4)	47 (11.9)	.16	
Neither agree nor disagree	101 (27.7)	90 (22.8)	.12	
Disagree	142 (39.0)	158 (40.0)	.78	
Completely disagree	51 (14.0)	86 (21.8)	.005	
The spouse should be routinely screened for sexual function when a male or female patient is diagnosed with sexual dysfunction				
Totally agree	91 (25.0)	142 (35.9)	.001	<.001
Agree	180 (49.5)	194 (49.1)	.93	
Neither agree nor disagree	68 (18.7)	39 (9.9)	<.001	
Disagree	20 (5.5)	11 (2.8)	.06	
Completely disagree	5 (1.4)	9 (2.3)	.36	

Abbreviation: NA, not applicable.

^a^ χ^2^ test or Fisher exact test for categorical variables was used as appropriate.

As shown in Table 3, there were statistically significant differences in all 4 practice patterns between groups with knowledge scores of 6 points or more vs fewer than 6 points. Since all the knowledge and practice patterns could be divided into male and female aspects, we further subdivided the knowledge and practice pattern questions into male (questions K1-K6 and P1-P2) and female (questions K7-K8 and P3-P4) categories. Mosaic plots were constructed to explore the distributions and associations between knowledge and practices related to male and female sexual dysfunction (Figure). Most participants had knowledge on male sexual dysfunction (540 participants [71.1%] answered correctly at least 4 of 6 questions on male sexual health), but they lacked knowledge on female sexual dysfunction (490 participants [64.5%] answered both questions on female sexual health incorrectly). Additionally, the proportions of participants who reported knowing and using guidelines to guide clinical work were positively associated with their knowledge scores for all 4 guidelines for male sexual dysfunction and female sexual dysfunction. Among individuals with passing knowledge scores, 232 participants (58.7%) reported knowledge and application of the guidelines for diagnosis and treatment of PE and 162 participants (41.0%) reported knowledge and application of the guidelines for the diagnosis and treatment of ED, which were significantly higher than rates among participants with nonpassing knowledge scores (PE: 140 participants [38.5%]; ED: 78 participants [21.4%]), while only a few urologists and andrologists could manage female sexual dysfunction following guidelines, although the proportions were higher in the group with passing knowledge (38 participants [9.6%]) compared with those with less knowledge (5 participants [1.4%]).

**Table 3.** Associations Between Practice Patterns and Knowledge Among Respondents

Question	Knowledge score, No. (%)		*P* value^a^
	<6 Points (n = 364)	≥6 Points (n = 395)	
P1: Do you have knowledge on the 2014 version of the International Society of Sexual Medicine’s guidelines for the diagnosis and treatment of premature ejaculation?			
I know and use it to guide clinical work	140 (38.5)	232 (58.7)	<.001
I know it, but I have not used it in clinical practice	141 (38.7)	135 (34.2)	
I have heard about it, but I do not know the contents	64 (17.6)	28 (7.1)	
I have no idea	19 (5.2)	0	
P2: Do you have knowledge on the 2018 version of the American Urological Association’s guidelines for the diagnosis and treatment of erectile dysfunction?			
I know and use it to guide clinical work	78 (21.4)	162 (41.0)	<.001
I know it, but I have not used it in clinical practice	158 (43.4)	184 (46.6)	
I have heard about it, but I do not know the contents	98 (26.9)	44 (11.1)	
I have no idea	30 (8.2)	5 (1.3)	
P3: Do you have knowledge on the ACOG Practice Bulletin clinical management guidelines for obstetrics/gynecology, No. 119/213, female sexual dysfunction?			
I know and use it to guide clinical work	5 (1.4)	38 (9.6)	<.001
I know it, but I have not used it in clinical practice	44 (12.1)	136 (34.4)	
I have heard about it, but I do not know the contents	145 (39.8)	129 (32.7)	
I have no idea	170 (46.7)	92 (23.3)	
P4: Do you have knowledge on the *DSM-5* classification of female sexual dysfunction?			
I know and use it to guide clinical work	8 (2.2)	30 (7.6)	<.001
I know it, but I have not used it in clinical practice	32 (8.8)	113 (28.6)	
I have heard about it, but I do not know the contents	150 (41.2)	151 (38.2)	
I have no idea	174 (47.8)	101 (25.6)	

Abbreviations: ACOG, American College of Obstetricians and Gynecologists; *DSM-5*, *Diagnostic and Statistical Manual of Mental Disorders* (Fifth Edition); P, practice question.

^a^ χ^2^ test or Fisher exact test for categorical variables was used as appropriate.

**Figure.**
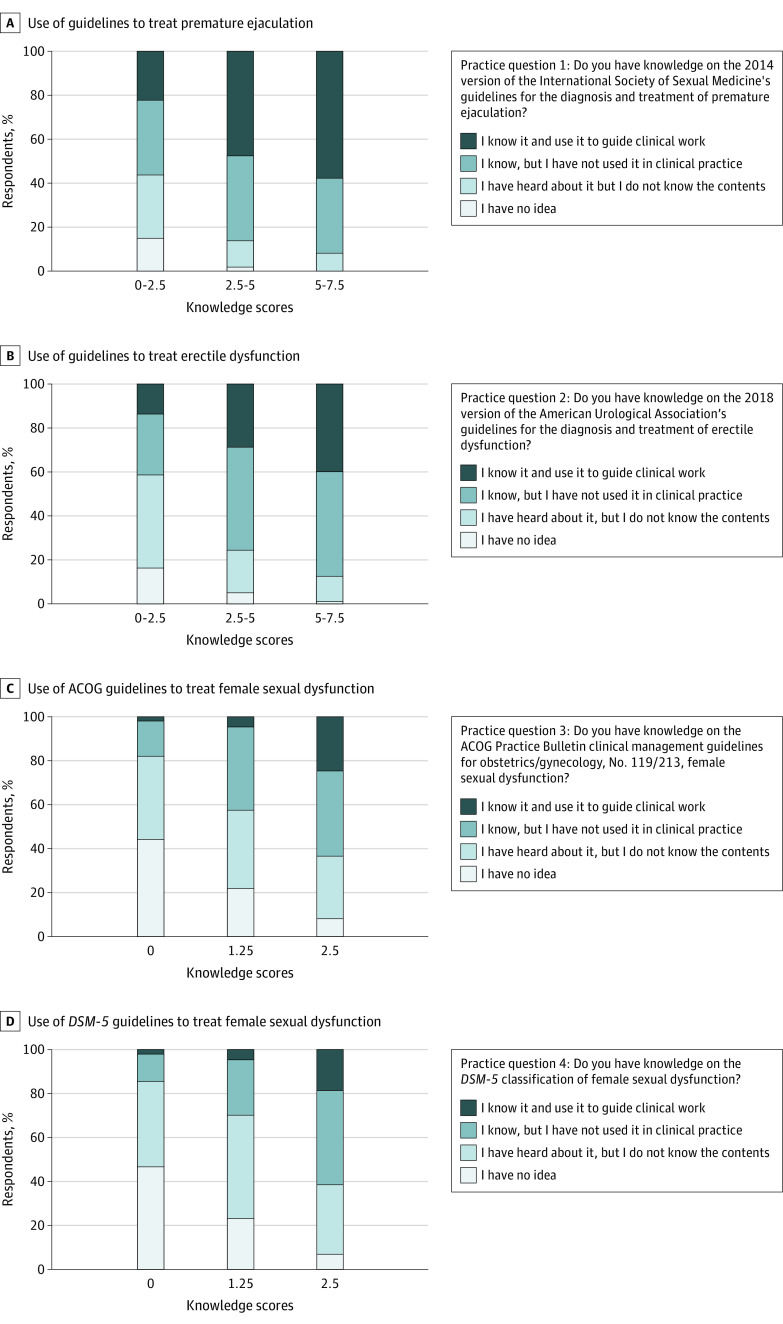
Associations Between Knowledge and Practice of Male and Female Sexual Function Following Clinical Guidelines ACOG indicates American College of Obstetricians and Gynecologists; *DSM-5*, *Diagnostic and Statistical Manual of Mental Disorders* (Fifth Edition).

A total of 569 participants (75.0%) felt confident about listening to and handling male patients’ sexual issues (ie, answered almost always or often), while only 274 participants (36.1%) reported feeling similarly confident on issues relating to female patients’ sexual issues. Some potential reasons for difficulties in managing sex issues are listed in Table 4. For the difficulties in dealing with male sexual issues, the main difficulties included lacking of effective treatment methods and drugs (391 participants [51.5%]), not having enough specific experience (359 participants [47.3%]) and lacking knowledge in the field (319 participants [42.0%]). Regarding the difficulties in dealing with female sexual issues, the most commonly reported problems were lacking of knowledge in the field (518 participants [68.2%]) and not having enough specific experience (432 participants [56.9%]). Detailed results are presented in Table 4.

**Table 4.** Main Difficulties Reported by the Respondents With Regard to Dealing With Sexual Health Issues

Question	Respondents, No. (%)
Do you have confidence in listening to and managing male patients’ sexual issues?	
Almost always	170 (22.4)
Often	399 (52.6)
Sometimes	155 (20.4)
Rarely	33 (4.3)
Never	2 (0.3)
What are the main difficulties you have experienced in listening to and managing male patients’ sexual issues?	
Lack of knowledge in this field	319 (42.0)
I do not have enough time	243 (32.0)
I do not have enough specific experience	359 (47.3)
I feel embarrassed to discuss it	64 (8.4)
Patients feel embarrassed to discuss it	270 (35.6)
Unfavorable clinical environment	255 (33.6)
Lack of effective treatment methods and drugs	391 (51.5)
I can deal with it well	126 (16.6)
Do you have confidence in listening to and managing female patients’ sexual issues?	
Almost always	89 (11.7)
Often	185 (24.4)
Sometimes	188 (24.8)
Rarely	220 (29.0)
Never	77 (10.1)
Main difficulties you have experienced in listening to and managing female patients’ sexual issues?	
Lack of knowledge in this field	518 (68.2)
I do not have enough time	107 (14.1)
I do not have enough specific experience	432 (56.9)
I feel embarrassed to discuss it	183 (24.1)
Patients feel embarrassed to discuss it	348 (45.8)
Unfavorable clinical environment	217 (28.6)
Lack of effective treatment methods and drugs	366 (48.2)
Beyond the scope of my specialty	186 (24.5)
I can deal with it well	36 (4.7)

## Discussions

This multicenter survey study among urologists and andrologists from different regions in China developed representative estimates of knowledge, attitudes, and practice patterns regarding male and female sexual dysfunction in China. To our knowledge, this is the first study to investigate these aspects among urologists and andrologists in China.

Only 395 urologists and andrologists (52.0%) scored at least 6 points on the knowledge test, out of a total score of 10 points, indicating a limited overall knowledge of sexual dysfunction, especially for female sexual dysfunction, and more than half of the participants answered the 2 knowledge questions on treatment of female sexual dysfunction incorrectly. Our findings also suggested that knowledge was associated with attitudes and practice patterns for management of sexual dysfunction. Urologists and andrologists with passing knowledge on sexual dysfunction expressed more positive attitudes toward addressing and managing sexual function issues and also felt that sexual issues among female patients deserved attention. Similarly, passing knowledge on sexual dysfunction was also associated with use of sexual dysfunction–related guidelines to guide clinical work. It is worth noting that most participants lacked passing knowledge on female sexual dysfunction and rarely used clinical guidelines of female sexual dysfunction to guide their clinical practices.

Generally, urologists and andrologists mainly focus on specific aspects of male sexual dysfunction, such as erectile dysfunction, orgasmic dysfunction, and premature ejaculation. Nevertheless, female sexual dysfunction is also prevalent and is associated with male sexual performance.^21^ According to previous studies, female sexual function parameters were significantly associated with their male partners’ erectile function and sexual desire.^8,15,21^ A previous study has found that 55% of female partners of men with ED reported 1 or more female sexual dysfunctions. Most male patients with sexual dysfunction (91%) presenting to the clinic were encouraged to do so by their female partners.^15^ Additionally, female sexual dysfunction was negatively associated with the success of the male partner’s treatment for sexual dysfunction.^19,22^ As a result, urologists and andrologists must be cognizant of not only the management of male sexual dysfunction but also female sexual dysfunction for proper diagnosis and treatment in clinical practice.

However, female patients are less likely to share information about sexual issues with urologists and andrologists in the context of traditional Chinese culture, especially to male urologists and andrologists, and few urologists and andrologists in China are female (only 1.3% of participants in this survey study were female). There are almost no professional female sexual medicine physicians or psychologists or clinics in China, and the status of screening and management of female sexual issues by obstetricians and gynecologists in China is also unsatisfactory.^14^ Although urologists and andrologists may not be the clinicians of choice for female individuals with sexual dysfunction issues, urologists and andrologists should still take the time to perform a brief sexual assessment of female sexual function when treating with their male partners with sexual dysfunction to assess possible reciprocal influences between them. A comprehensive diagnosis and treatment pattern, including obstetricians and gynecologists, psychologists, and urologists and andrologists, may be more beneficial for management of sexual dysfunction, especially for coexistent sexual dysfunction in couples. Improvement on knowledge and positive attitudes is necessary not only for obstetricians and gynecologists and psychologists, but also for urologists and andrologists.

This survey study found that level of knowledge on sexual dysfunction was associated with attitudes and practice patterns for urologists and andrologists in management of sexual issues in China. Additionally, urologists and andrologists reported that lacking sexual dysfunction–related knowledge was a core difficulty in managing these conditions, demonstrating the need for continuing education in this field. Internationally, the state of training and continuing education in sexual medicine is mostly considered inadequate.^12,13,23^ Increased efforts are being made to improve the overall quality of training in sexual medicine by discipline and country to ensure high-quality sexual medicine education.^24^ For urologists and andrologists, adequate training and continuing education could improve their knowledge, attitudes, practices, and confidence in management of sexual dysfunction for both male and female patients.

### Limitations

This study has several limitations. First, the quota sampling method was applied in this survey, which could lead to biased sampling. Second, only 759 participants responded to this survey, resulting in a relatively low response rate of 45%, which could lead to nonresponse bias. Third, only a subset of sexual dysfunction–related knowledge and attitudes were captured among a subset of urology and andrology practices enrolled in this survey, and we were not able to capture all important aspects of knowledge, attitudes, and practices. Fourth, the data collected in the study relied on self-report rather than practice observations and therefore may not fully reflect current clinical practice.

## Conclusions

The results of this survey study suggest that urologists and andrologists in China generally lacked knowledge about sexual function and that lacking of knowledge was associated with urologists’ and andrologists’ attitudes and practice patterns. Additionally, urologists and andrologists in China seriously lacked knowledge of female sexual function, an issue of serious concern, given that couples’ sexual issues are closely related and largely affect each other. We recommend that urologists and andrologists receive adequate training in sexual medicine, an important strategy for meeting the future sexual and reproductive health needs of everyone.

## Notes

Supplement 1. **eAppendix.** Supplementary Methods

Supplement 2.
Data Sharing Statement
