# Supplementary material for: Knowledge, Attitudes, and Practice Patterns Relating to Sexual Dysfunction Among Urologists and Andrologists in China
Source: JAMA Netw Open. 2023 Jan 12;6(1):e2250177. doi: 10.1001/jamanetworkopen.2022.50177 (PMC9857643; doi:10.1001/jamanetworkopen.2022.50177)
Supplement: Supplement 1. — eAppendix. Supplementary Methods [file jamanetwopen-e2250177-s001.pdf]

# Supplemental Online Content

Tang D, Zhang Y, Zhang W, et al. Knowledge, attitudes, and practice patterns relating to sexual dysfunction among urologists and andrologists in China. *JAMA Netw Open*. 2023;6(1):e2250177. doi:10.1001/jamanetworkopen.2022.50177

**eAppendix.** Supplementary Methods

This supplemental material has been provided by the authors to give readers additional information about their work.

© 2023 Tang D et al. *JAMA Network Open.*

**eAppendix.** Supplementary Methods

***Sampling Method***

At present, a comprehensive list of all licensed physicians in China was not available. According to China Function Statistics Yearbook (National Function Commission of the People's Republic of China, http://www.nhc.gov.cn/), the Eastern region refers to the economically developed provinces, while the Midwest region refers to the less economically developed regions. In addition, urban areas refers to municipalities directly under the central government and prefecture-level cities, whereas rural areas refers counties and county-level cities. Consequently, a two-stage quota sampling method was applied to achieve a nationally representative sample. The first step was the sampling of hospitals. Although the ratio of distributed number of hospitals in rural to urban areas is 4:1 in China, it is noteworthy that the vast majority of Urologists/Andrologists in China are employed in secondary or tertiary hospitals (In China, hospitals are categorised into three: primary, secondary and tertiary hospital). Therefore, we selected 100 secondary or tertiary medical centers according to the distribution of secondary or tertiary medical centers in China based on rural/urban and Midwest/East parts (the ratio of rural/urban is roughly 1:1; the ratio of Midwest/East is roughly 6:4; the data come from the China Function Statistics Yearbook). One hundred secondary/tertiary medical centers registered in China Sexology Association were randomly selected, including 30 from Midwestern urban areas, 30 from eastern urban areas, 20 from Midwestern rural areas and 20 from eastern rural areas were selected. The second step was sampling physicians. In the selected 100 hospitals, the ratio of physicians were designed to be invited according the distribution of physicians in China (the ratio of rural/urban is roughly 5:3; the ratio of Midwest/East is roughly 9:7; the data come from the China Function Statistics Yearbook, National Function Commission of the People's Republic of China, http://www.nhc.gov.cn/). Eventually, a total of 1600 physicians were invited to participant in this survey from the 100 selected hospitals, including 500 from Midwestern urban areas, 500 from eastern urban areas, 400 from Midwestern rural areas and 200 from eastern rural areas.

***Scoring Method***

In order to quantitatively explore the association among knowledge, attitudes, and practice patterns related to sexual dysfunction, combining with previous articles (Li G, et al. JAMA Netw Open 2021, e2110695), we decided to adopt ten-mark system. For "Knowledge", each question of the knowledge question with a total of eight item scored 1.25. For each knowledge question, the three answers were distributed as agree, neutral, and disagree. The answer of"agree"indicating mastery of the corresponding knowledge, would be scored 1.25, while"neutral" or "disagree"would be scored 0 as lacking the corresponding knowledge. Similarly, for "Attitude", the full score for each question was set as 2, so as to accord with the ten-mark system with the 5 questions in total. A 5-point Likert scale was applied in this part, and was scored as follows: totally agree (2), agree (1.5), neither agree nor disagree (1.0), disagree (0.5), and completely disagree (0), according to the previous article (Li G, et al. JAMA Netw Open 2021,e2110695).

© 2023 Tang D et al. *JAMA Network Open.*

**eAppendix. Survey questionnaire (English version)**

## Survey on the knowledge, attitude and practice patterns of Urologist/Andrologist in sexual function

Dear doctors from Urology and Andrology:

Thanks for your attention on our e-mails. On behalf of Professor Xiansheng Zhang, and Professor Hui Jiang from the China Sexology Association, we invited you to participate in the present survey intended to explore the knowledge, attitudes, and practice patterns related to sexual function among urologists/andrologists (UR/ANDs) in China. We contact you to participate in the survey as you are involving in the clinical practice of urology and andrology. Our research survey includes 29 questions including your demographic characteristics, knowledge, attitude and practice patterns related to sexual function both in male and female, and may occupy you about 2-10 min to complete it. The results of this survey will be analyzed further to understand Chinese UR/ANDs' knowledge, attitude, and practice patterns in dealing with sexual dysfunctions both in male and female.

The survey is conducted using Wenjuan Star. All data will be stored in a secure drive. Data will only be accessible to the study team. So, your private information involved in our survey is absolutely safe. During your participation, you could stop or quit the survey whenever if you feel uncomfortable on some questions.

There is no personal benefit from your participation in the survey. And the results

© 2023 Tang D et al. *JAMA Network Open.*

gained from the survey may benefit future patients with sexual dysfunctions. More importantly, it will give us a specific direction on the training for sexual medicine in the future. If you have any questions about the survey or other related other questions, please don't hesitate to contact the study coordinator Dr. Dongdong Tang (tangdongdong@ahmu.edu.cn). If you agree to participate in our study, please enter your name and date in the box below to indicate informed consent. (e.g., Li Ping, May 1, 2022)

( ________________________________ )

Thank you for your time and consideration.

Xiansheng Zhang, MD, PhD

Professor, Urology and Andrology

Vice-chairman, Global Chinese Andrology and Sexology Association

Vice-director, China Sexology Association

Director, China Sexology Association (Anhui Branch)

Hui Jiang, MD, PhD

Professor, Urology and Andrology

Chairman, Global Chinese Andrology and Sexology Association

Director, China Sexology Association

E-mails: zhangxiansheng@ahmu.edu.cn, jianghui@bjmu.edu.cn.

© 2023 Tang D et al. *JAMA Network Open.*

**Survey on the knowledge, attitude and practice patterns of Urologist/Andrologist in sexual function**

1 Your age?

- 20-35y
- 36-50y
- ≥51y

2 Your sex?

- Male
- Female

3 What is your educational level?

- Bachelor or below
- Master degree
- Doctorate

4 What is your practice setting?

- Tertiary public hospital
- Second public hospital

5 What is your professional title?

- Senior grade
- Medium grade
- Junior grade

6 How long have you majored in urology/andrology?

- <5y
- 5-15y
- >15y

7 What is your sub-specialty type?

- Traditional medicine
- Western medicine

8 What is the ratio of sexual dysfunction in your patients?

- <25%
- 25-50%

© 2023 Tang D et al. *JAMA Network Open.*

- 50-75%
- >75%

**Knowledges relating to sexual function.**

9(K1) Are you familiar with the definition of PE?

- Know it completely
- I am not sure
- I don't know it

10(K2) What is your opinion on the treatment of selective dorsal neurotomy for PE?

- Agree
- Disagree
- Neutral

11(K3) What is your opinion on the fasting state measurement of the testosterone level for ED?

- Agree
- Disagree
- Neutral

12(K4) What is your opinion on the prognostic role of ED in the cardiovascular disease event?

- Agree
- Disagree
- Neutral

13(K5) Do you have knowledge on the methods and related risk of PDE5i in the treatment for ED?

- Agree
- Disagree
- Neutral

14(K6) What is your opinion on the venous surgery in the treatment for ED?

- Agree
- Disagree
- Neutral

© 2023 Tang D et al. *JAMA Network Open.*

15(K7) What is your opinion on the use of Sildenafil for female sexual dysfunction?

- Agree
- Disagree
- Neutral

16(K8) What is your opinion on the use of Flibanserin for treating hyposexuality disorder in premenopausal women without depression”?

- Agree
- Disagree
- Neutral

**Attitudes relating to sexual function.**

17(A1) Sexual life is private and should not be interfered.

- Totally agree
- Agree
- Neither agree nor disagree
- Disagree
- Completely disagree

18(A2) I am interested in providing sex counseling or sexual function care to patients.

- Totally agree
- Agree
- Neither agree nor disagree
- Disagree
- Completely disagree

19(A3) I do not think female sexual function issues are important or “priority diseases”.

- Totally agree
- Agree
- Neither agree nor disagree
- Disagree
- Completely disagree

20(A4) Screening and managing female sexual function issues is more of the

© 2023 Tang D et al. *JAMA Network Open.*

responsibility of obstetricians and gynecologists than of urologists or sexologist.

- Totally agree
- Agree
- Neither agree nor disagree
- Disagree
- Completely disagree

21(A5) The spouse should be routinely screened for sexual function when the male or female was diagnosed for sexual dysfunction.

- Totally agree
- Agree
- Neither agree nor disagree
- Disagree
- Completely disagree

**Practice patterns relating to sexual function.**

22(P1) Do you have knowledge on the 2014 version International Society of Sexual Medicine's guidelines for the diagnosis and treatment of premature ejaculation (PE)

- I know and used it to guide clinical work
- I know, but I have not used it in clinical practice
- I've heard about it but I don't know the contents
- I have no idea

23(P2) Do you have knowledge on the 2018 version American Urological Association’s guidelines for the diagnosis and treatment of erectile dysfunction (ED)

- I know and used it to guide clinical work
- I know, but I have not used it in clinical practice
- I've heard about it but I don't know the contents
- I have no idea

24(P3) Do you have knowledge on the ACOG Practice Bulletin clinical management guidelines for Obstetrics/Gynecology, No.119/213, Female Sexual Dysfunction?

- I know and used it to guide clinical work
- I know, but I have not used it in clinical practice

© 2023 Tang D et al. *JAMA Network Open.*

- I've heard about it but I don't know the contents
- I have no idea

25(P4) Do you have knowledge on the DSM-5 classification of female sexual dysfunction?

- I know and used it to guide clinical work
- I know, but I have not used it in clinical practice
- I've heard about it but I don't know the contents
- I have no idea

**Main difficulties to dealing with the sexual function issues.**

26 Do you have confidence in listening and managing male patients' sexual function issues?

- Almost every time
- Often
- Sometimes
- Rarely
- Never

27 Main difficulties you have experienced in listening and managing male patients' sexual function issues?

- Lack of knowledge in this field
- I don't have enough time
- I don't have enough specific experience
- I feel embarrassed to discuss it
- Patients feel embarrassed to discuss it
- Unfavorable clinical environment
- Lack of effective treatment methods and drugs
- I could deal with it well

28 Do you have confidence in listening and managing female patients' sexual function issues?

- Almost every time
- Often

© 2023 Tang D et al. *JAMA Network Open.*

- Sometimes
- Rarely
- Never

29 Main difficulties you have experienced in listening and managing female patients' sexual issues?

- Lack of knowledge in this field
- I don't have enough time
- I don't have enough specific experience
- I feel embarrassed to discuss it
- Patients feel embarrassed to discuss it
- Unfavorable clinical environment
- Lack of effective treatment methods and drugs
- Beyond the scope of my specialty
- I could deal with it well

© 2023 Tang D et al. *JAMA Network Open.*
