# Supplementary material for: Knowledge, Attitudes, and Practice Patterns Relating to Sexual Dysfunction Among Urologists and Andrologists in China
Source: JAMA Netw Open. 2023 Jan 12;6(1):e2250177. doi: 10.1001/jamanetworkopen.2022.50177 (PMC9857643; doi:10.1001/jamanetworkopen.2022.50177)
Supplement: Supplement 2. — Data Sharing Statement [file jamanetwopen-e2250177-s002.pdf]

Data Sharing Statement

Tang. Knowledge, Attitudes, and Practice Patterns Relating to Sexual Dysfunction Among Urologists and Andrologists in China. *JAMA Netw Open*. Published January 12, 2023. doi:10.1001/jamanetworkopen.2022.50177

Data
Data available: Yes
Data types: Data dictionary
How to access data: The datasets used and/or analysed during the current study are available from the corresponding author on reasonable request.
When available: With publication

Supporting Documents
Document types: None

Additional Information
Who can access the data: The datasets used and/or analysed during the current study are available from the corresponding author on reasonable request for researchers on this topic.
Types of analyses: For researchers on this topic
Mechanisms of data availability: With a signed data access agreement
